# A novel platform for electromagnetic navigated ultrasound bronchoscopy (EBUS)

**DOI:** 10.1007/s11548-015-1326-7

**Published:** 2015-11-28

**Authors:** Hanne Sorger, Erlend Fagertun Hofstad, Tore Amundsen, Thomas Langø, Håkon Olav Leira

**Affiliations:** Department of Thoracic Medicine, St. Olavs Hospital, Postboks 3250, Sluppen, 7006 Trondheim, Norway; Department of Circulation and Medical Imaging, Faculty of Medicine, Norwegian University of Science and Technology (NTNU), AHL-senteret, Prinsesse Kristinas gate 3, Trondheim, Norway; Department Medical Technology, SINTEF, Technology and Society, Trondheim, Norway; Department of Medicine, Levanger Hospital, Nord-Trøndelag Health Trust, Levanger, Norway

**Keywords:** Electromagnetic navigation, Endobronchial ultrasound, Convex probe endobronchial ultrasound (CP-EBUS), Navigated ultrasound bronchoscopy, Navigated EBUS, Multimodal image fusion

## Abstract

**Purpose:**

Endobronchial ultrasound transbronchial needle aspiration (EBUS-TBNA) of mediastinal lymph nodes is essential for lung cancer staging and distinction between curative and palliative treatment. Precise sampling is crucial. Navigation and multimodal imaging may improve the efficiency of EBUS-TBNA. We demonstrate a novel EBUS-TBNA navigation system in a dedicated airway phantom.

**Methods:**

Using a convex probe EBUS bronchoscope (CP-EBUS) with an integrated sensor for electromagnetic (EM) position tracking, we performed navigated CP-EBUS in a phantom. Preoperative computed tomography (CT) and real-time ultrasound (US) images were integrated into a navigation platform for EM navigated bronchoscopy. The coordinates of targets in CT and US volumes were registered in the navigation system, and the position deviation was calculated.

**Results:**

The system visualized all tumor models and displayed their fused CT and US images in correct positions in the navigation system. Navigating the EBUS bronchoscope was fast and easy. Mean error observed between US and CT positions for 11 target lesions (37 measurements) was $2.8\pm 1.0$ mm, maximum error was 5.9 mm.

**Conclusion:**

The feasibility of our novel navigated CP-EBUS system was successfully demonstrated. An EBUS navigation system is needed to meet future requirements of precise mediastinal lymph node mapping, and provides new opportunities for procedure documentation in EBUS-TBNA.

## Introduction

Lung cancer is the second most frequent cancer worldwide, and five-year survival rates are only 10–15 % [1, 2]. The clinical challenge is early identification of patients with limited disease, who have a markedly better prognosis and should be offered curative treatment [3]. Metastatic involvement of mediastinal lymph nodes rules out surgery, and thorough mediastinal investigation is therefore the crucial point of the diagnostic work-up.

**Fig. 1:**
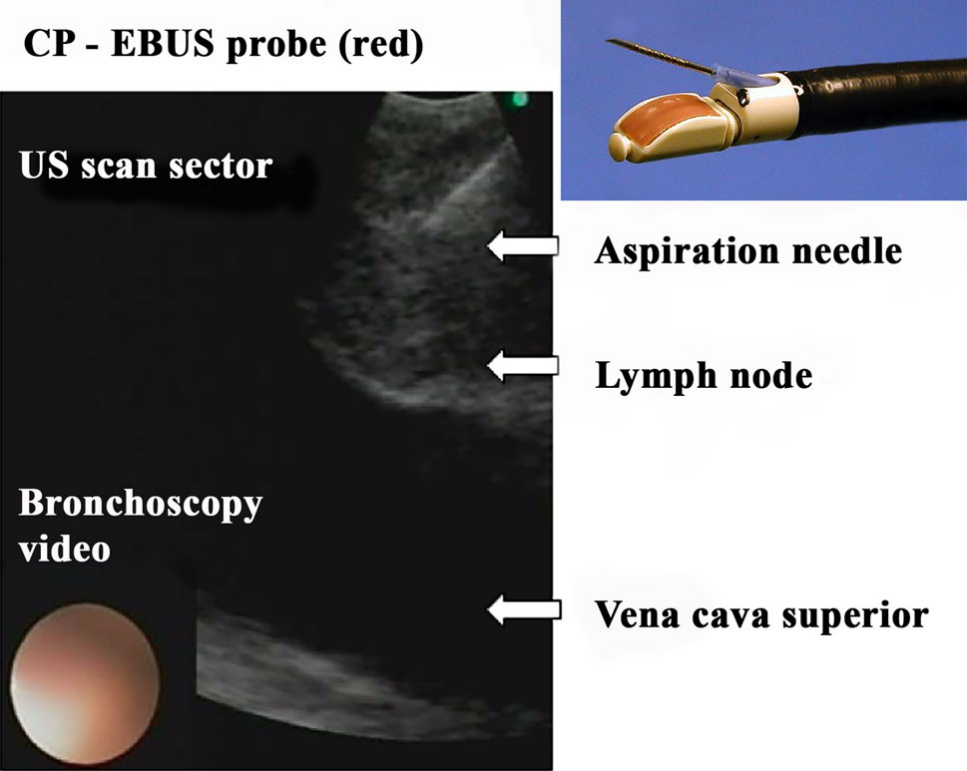
Convex probe endobronchial ultrasound (CP-EBUS) guiding real-time transbronchial fine-needle aspiration of a mediastinal lymph node. The sampling needle is visualized sonographically inside the lymph node. Video bronchoscopy is displayed simultaneously (*bottom left*). The transbronchial needle system emerges from the bronchoscope’s working channel just proximal to the CP-EBUS probe (*top right*)

Endobronchial ultrasound transbronchial needle aspiration (EBUS-TBNA) is now the method of choice for tissue confirmation of mediastinal lymph node metastasis [4–9], and helps avoid futile more invasive lung cancer staging procedures [10–15]. An ultrasound (US) probe in the tip of the bronchoscope allows visualization and guides fine-needle sampling of anatomical structures outside the tracheobronchial wall (Fig. 1).

For real-time TBNA guiding of lymph nodes positioned close to the central airways, the convex probe EBUS bronchoscope (CP-EBUS) is superior [16]. In CP-EBUS, a curvilinear US probe (normally 7.5 MHz, frequency range 5–12 MHz) scans a $60^{\circ }$ image sector parallel to the shaft of the bronchoscope (Fig. 1). Video bronchoscopy is displayed simultaneously on the US monitor and used for endobronchial inspection and localization of the approximate region of interest. The target lesion is identified sonographically, if necessary by using a water-filled disposable latex balloon attached to the US probe. Under direct US visualization, a transbronchial needle system is inserted through the working channel of the bronchoscope, advanced until emerging proximal to the US probe, and pushed through the bronchial wall into the target lymph node (Fig. 1). Using intermittent suction and agitation of the needle, a specimen is aspirated. After suction is released, the entire needle system is removed from the bronchoscope in one movement. The cytological material is deposited on a microscope slide for smear preparation. The number of needle aspirations per target can impact the diagnostic yield of CP-EBUS-TBNA, and three punctures are recommended [17].

Although the method’s sensitivity and diagnostic accuracy are excellent [11, 12, 15], CP-EBUS-TBNA is highly operator-dependent. The bronchoscopist’s ability to remember the spatial position of the target lymph node from preoperative images is crucial. Anatomical changes can also occur between preoperative imaging and the diagnostic bronchoscopy. Real-time correction with US is necessary, particularly due to respiratory movements. The drawbacks of 2D EBUS are that the field of view is small, the identification of pathological structures can be hard, and artifacts are common. Puncturing the wrong lymph node will have important clinical implications in lung cancer staging. Especially with less experienced operators and in patients with multiple adjacent lymph nodes in the same anatomical region, this is a real possibility. Close attention should therefore be paid to reduce intraoperative technical- and operator-related factors causing errors in CP-EBUS-guided sampling and clinical decision making.

In lung cancer staging, CP-EBUS-TBNA has previously targeted mainly lymph nodes that are pathologically enlarged ($>$10 mm) in the preoperative CT scan, and/or nodes that are positron emission tomography–computed tomography (PET–CT) positive. The clinical use of CP-EBUS-TBNA is currently shifting toward routine systematic mapping of even normal size (5–10 mm) lymph nodes below the detection size for PET–CT [7]. These nodes are technically more challenging to sample, and the clinical need for precise and effective guiding during CP-EBUS-TBNA is therefore increasing.

Advanced image-guiding technology, such as electromagnetic navigated bronchoscopy (ENB), can facilitate endoscopic access to mediastinal lymph nodes [18–23]. In ENB systems, intraoperative navigation is made possible by sensors for position tracking of the bronchoscope and/or sampling tools in an electromagnetic (EM) field generated around the patient in the bronchoscopy suite. Combined US and ENB for increased sampling precision during bronchoscopy has also been explored with commercially available systems, but only using the radial US probe (RP-EBUS) designed for the peripheral airways and without real-time tracking of the EBUS images [24, 25]. The improvement in diagnostic yield was not enough to justify widespread adoption of the combined technology.

Real-time position tracking of any EBUS bronchoscope (RP-EBUS and CP-EBUS) has not been available so far. Only a few research groups have addressed this issue. Luo and Mori have presented a research system for navigated RP-EBUS, trying to improve position control during sampling of lung tumors in the peripheral airways [26]. The system implies preoperative CT images, electromagnetic tracking (EMT), and position measurements from external sensors mounted on an US miniprobe and a flexible bronchoscope. The reported tracking accuracy (CT/EMT registration accuracy) was 2.6 mm in preclinical studies [26]. In 2011, Mori et al. presented another technical solution for navigated RP-EBUS based on CT–US calibration [27], reporting a total accuracy of 0.97 mm in a preclinical phantom experiment. This has yet to be validated in clinical studies.

Another system for multimodal image guiding in bronchoscopy was recently presented by Zang et al. [28]. Their prototype integrates data from a CP-EBUS bronchoscope into their own, previously developed virtual bronchoscopy (VBN) image-guided intervention system [29]. The operator uses the preoperative CT volume to define a diagnostic region of interest. An optimal airway navigation route to target is calculated in the VBN system, and 2D CP-EBUS images are synchronized with CT data intraoperatively. The system is able to reconstruct and depict the 3D CP-EBUS volume around the region of interest, but this requires user-selected CP-EBUS key frames in advance.

Our research group has previously developed software and hardware platforms for image-guided interventions [30–33]. In this paper, we present a prototype EM navigation system designed for CP-EBUS-TBNA of mediastinal lymph nodes, with intraoperative position tracking of the EBUS bronchoscope. The system fuses preoperative computed tomography (CT) and real-time 2D US images in our existing platform for ENB. Taking advantage of the high spatial resolution and 3D overview of preoperative CT combined with the temporal resolution of real-time 2D US, the hope is to overcome the disadvantages of each imaging method alone.

We suggest that EM navigated CP-EBUS and multimodal imaging may improve CP-EBUS-TBNA precision in lung cancer staging, shorten the procedure time, provide new possibilities for documentation, and serve as a learning tool for apprentice endoscopists. The purpose of this phantom study was to validate the preclinical feasibility of the navigated CP-EBUS experimental platform. The paper describes technical components, accuracy measurements, and possible future applications of the new technology.

## Materials and methods

### Experimental setup

**Fig. 2:**
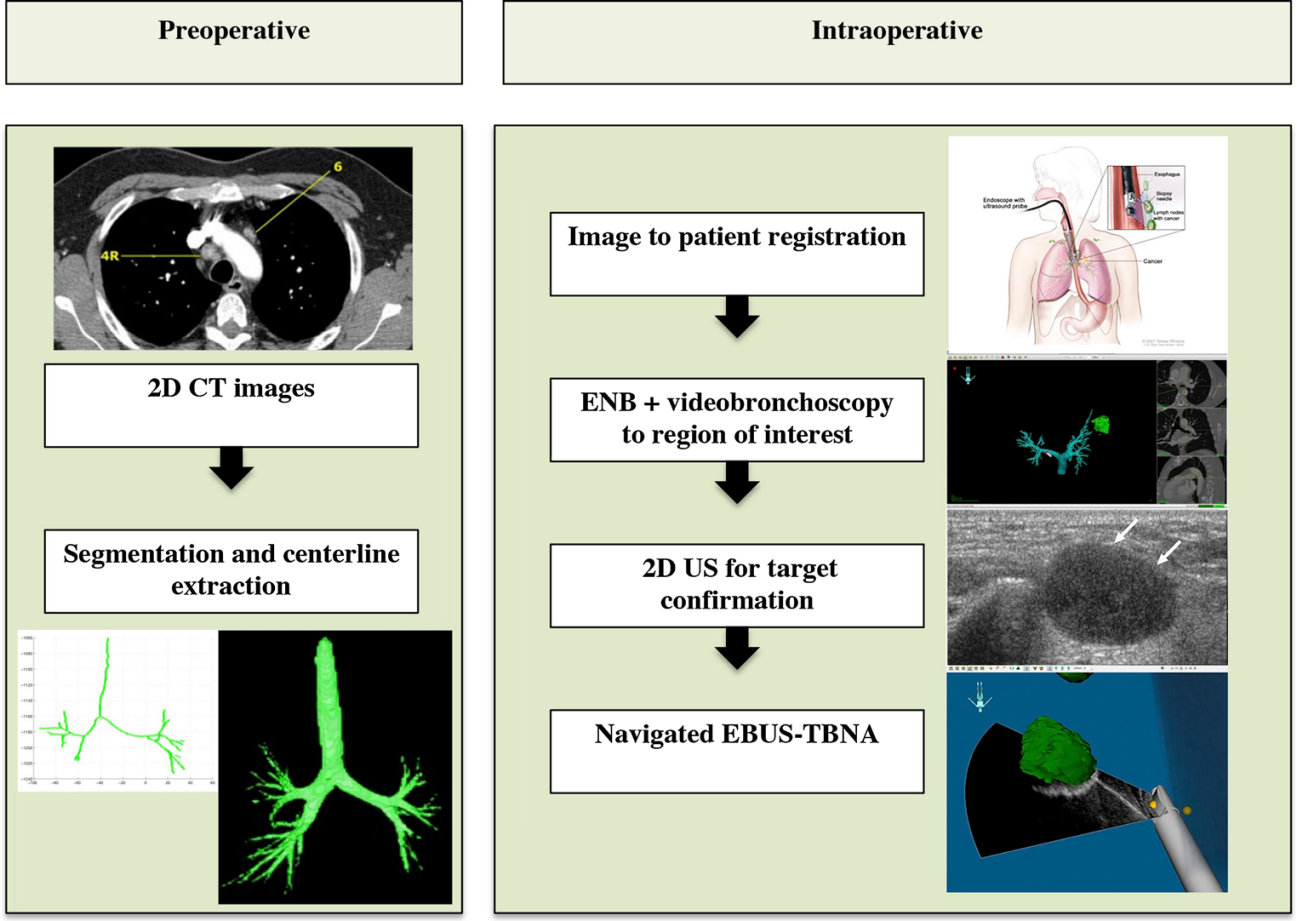
Schematic workflow of navigated convex probe endobronchial ultrasound (CP-EBUS) in the diagnosis of mediastinal lymph node metastasis in a fictive lung cancer case. (1) Mediastinal lymph nodes suspect of metastasis are identified in preoperative 2D CT and/or PET–CT images (*top left*), defining the region of interest for CP-EBUS-guided sampling. If a primary lung tumor is also visible, bronchoscopic sampling of the tumor is also considered; (2) the CT images are preprocessed. The target for sampling (lymph node, tumor) is segmented, and the airway centerline is extracted (*bottom left*); (3) image-to-patient centerline-based registration is performed in the operation room in the first phase of EBUS (*top right*, EBUS figure by Terese Winslow, Bronchoscopy, NCI Visuals Online, National Cancer Institute); (4) the level of the target lesion is identified by combined video bronchoscopy and electromagnetic (EM) position tracking of the CP-EBUS bronchoscope (EM navigation, *middle right*), (5) 2D EBUS (*middle right*) is used for target confirmation and visualization when the region of interest is approached and will aid the physician in deciding an optimal site for transbronchial needle aspiration (TBNA) (EM navigated CP-EBUS, *bottom right*), (6) TBNA from the target lymph node can be performed (*bottom right*). A cytology smear will reveal whether metastatic lung cancer is present

The navigated EBUS system is based on an existing navigation research platform, CustusX (SINTEF, Trondheim, Norway, http://www.custusx.org) [34]. Figure 2 shows the pre- and intraoperative workflow of EM navigated CP-EBUS. Main system components are:

*Computer and software*

- Desktop computer [AMD FX($^\mathrm{TM}$)-8350 Eight-Core Processor, 32 GB Memory, Ubuntu 12.04 64-bit]
- Research navigation software platform (CustusX)

*Bronchoscopy equipment*

- Flexible bronchoscope (Olympus BF Q160, Olympus, Tokyo, Japan) for ENB
- CP-EBUS bronchoscope (Olympus BF UC16OF, Olympus, Tokyo, Japan) for navigated EBUS
- Video processing unit (Olympus, CV-190, Olympus, Tokyo, Japan)
- Light source (Olympus CLV-190, Olympus, Tokyo, Japan)
- Ultrasound processor (Olympus EUS Exera EU-C60, Olympus, Tokyo, Japan)

*Electromagnetic position tracking system*

- $\hbox {Aurora}^{\circledR }$ (Northern Digital Inc. (NDI), Waterloo, ON, Canada)
- Tracking sensor (Aurora 6DOF Probe, Straight Tip, Standard, Northern Digital Inc., Waterloo, ON, Canada)

*Phantom*

- An in-house prepared gelatin-based airway phantom, containing silicone tumor models/targets

### Navigation platform

The research and development system for intraoperative image guiding upon which the navigated EBUS platform is based, CustusX, is open source (http://www.custusx.org). The system is used by researchers and commercial vendors both internationally and as part of a national research infrastructure. CustusX is already applied in other clinical areas, f.i. neurosurgery, laparoscopy, endovascular therapy, and lung endoscopy [30–34].

CustusX has modules for importing preoperative radiology images and for real-time streaming and visualization of intraoperative images such as US. The navigation system combines information from the pre- and intraoperative images with information from EM or optical position tracking systems reporting the position and orientation of surgical instruments, in this case the bronchoscope with EMT (Fig. 2) [32, 34].

Image preprocessing involves extracting anatomical structures (e.g., airways, tumor, lymph nodes) and line segments representing the airway centerline (Fig. 2). A time-saving automatic airway and centerline segmentation method was recently integrated in CustusX [35]. Tumors and lymph nodes are semiautomatically or manually segmented in external software (OsiriX [36] and ITK-SNAP [37]).

The navigation platform allows several opportunities for graphical user interface (GUI) organization [34], e.g., four panels with axial, coronal, and sagittal (ACS) CT views, and a main window displaying a 3D view of the bronchial tree or airway centerline [32]. A model of the tracked tool tip is integrated. Target lesions are outlined by segmentation. While the surface model and volume rendering provides overview, the orthogonal slices ACS, or alternatively a plane following the position and orientation of the tracked tool (any plane), provide important details from the original preoperative images. Movement of the EBUS bronchoscope shifts the GUI slices accordingly in real time. The boundaries of the US cone in the corresponding plane are visualized as a white sector in the CT panel and main window.

The exact location of the bronchoscope during sampling (in relation to image modality) and the relevant anatomical area covered during the procedure are documented and stored in the navigated EBUS system, and the whole procedure may later be replayed.

### Bronchoscopy equipment

**Fig. 3:**
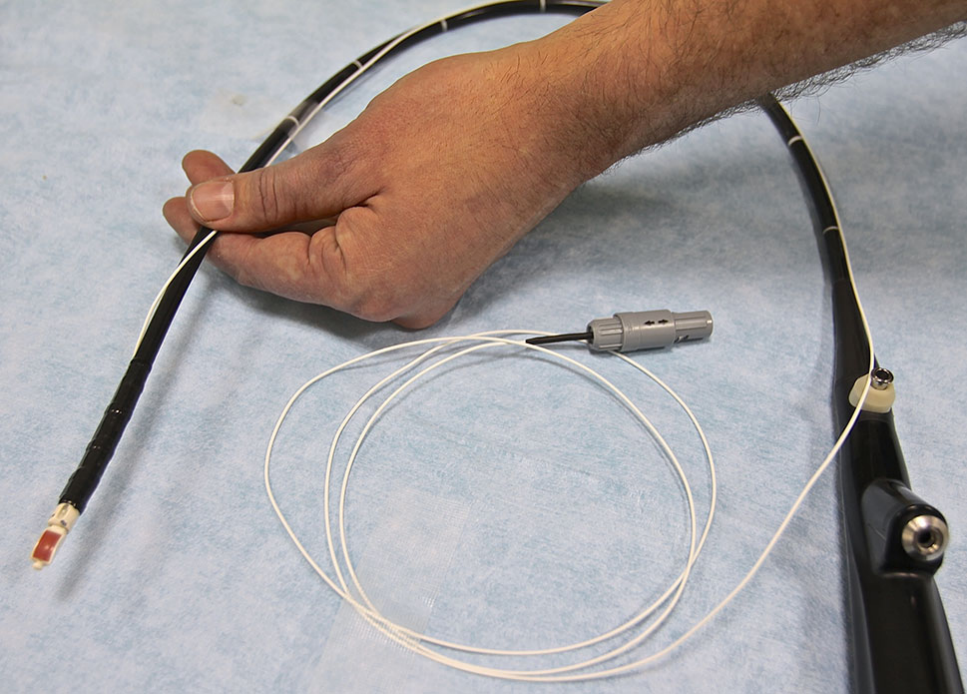
Prototype convex probe endobronchial ultrasound (CP-EBUS) bronchoscope with electromagnetic sensor integrated for position tracking

**Fig. 4:**
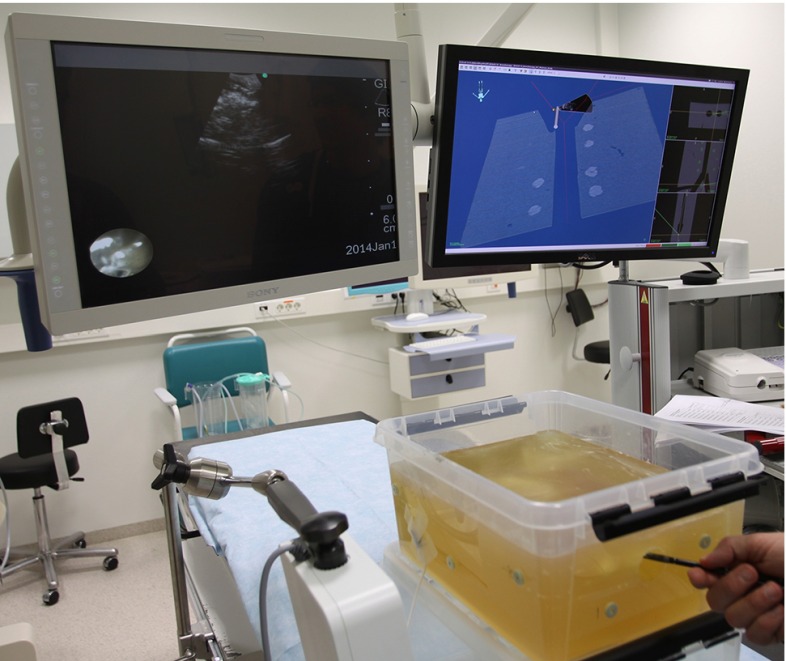
OR setup and interface during navigated convex probe endobronchial ultrasound (CP-EBUS) of a phantom model

Our research group has previously modified a flexible bronchoscope, integrating an EMT sensor for position tracking [38]. We have also made a prototype traceable US bronchoscope (2.0 mm work channel) (Fig. 3). A six degrees-of-freedom (DOF) EMT sensor has been attached parallel to the bending section, making it possible to track the movement of the EBUS bronchoscope in 3D space inside the patient/model. The EBUS bronchoscope is connected to the bronchoscopy monitor, a video processing unit, light source, and US processor. The combined navigation/US scene is displayed on a separate monitor (Fig. 4).

### Electromagnetic tracking

The position of the EMT sensor and hence the US transducer of the EBUS bronchoscope is measured using the Aurora Electromagnetic Tracking System$^{\circledR }$. The tracking system consists of a system control unit, maximum of four system interface units for position sensor inputs, and a field generator that generates an electromagnetic tracking volume. The sensors sample positions at a frequency of 40 Hz, with an accuracy of 0.48 mm, according to the manufacturer [39].

### Phantom

**Fig. 5:**
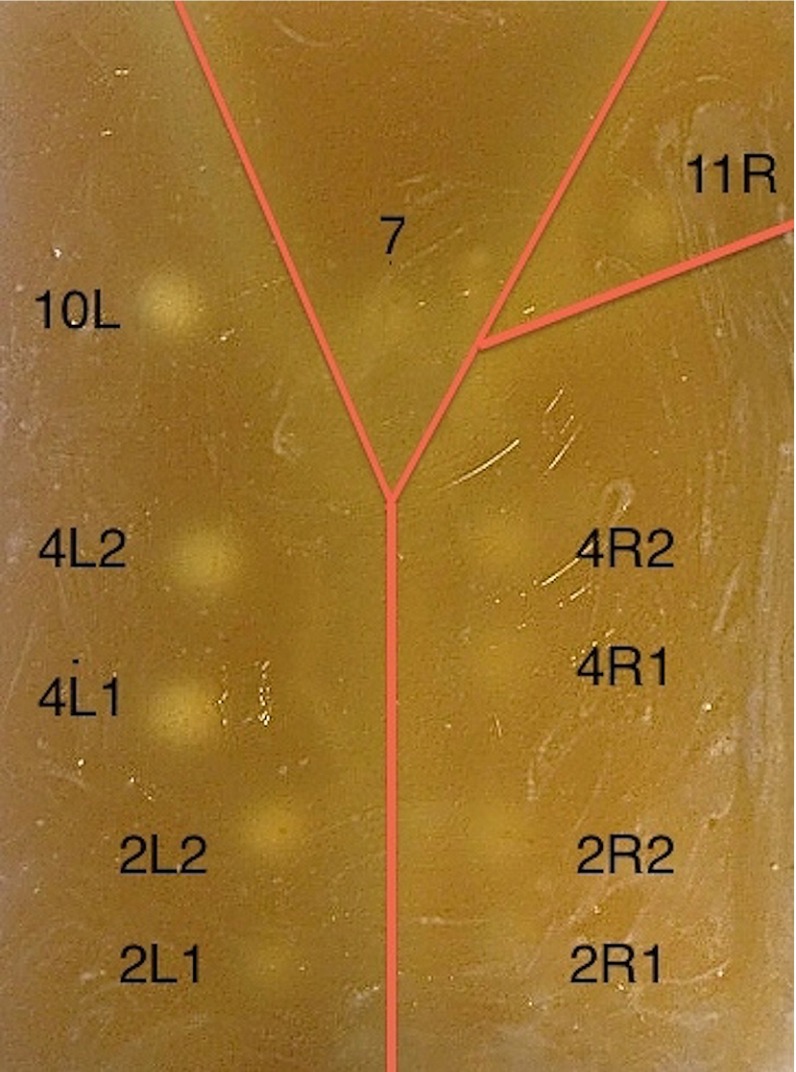
Gelatin-based airway phantom used for endoscopy. The course and divisions of the phantom airways are indicated by a *red line*. All tumor models are assigned numbers according to their location in the phantom mediastinal space, and a letter indicating right (*R*) or left (*L*) side

For this navigated EBUS feasibility experiment, we prepared a gelatin-based airway phantom mimicking the trachea and main bronchi, containing silicone tumor models serving as targets (Fig. 5). The targets were positioned mimicking mediastinal lymph node stations assigned 2, 4, 7, 10L, and 11R according to the IASLC nomenclature 7th edition [6]. Ten fiducial markers were evenly distributed on the phantom container, for the purpose of image registration. An EM positioning reference sensor was mounted on the phantom. CT of the phantom was performed preoperatively (Siemens Somatom Sensation, 64 slices, software syngo CT 2007S, VX68A, SL02P04, Windows 5.1), slice thickness 0.3 millimeters. CT images were imported into the navigation system. Tumor models were segmented using a thresholding method [35].

### Relationship between the coordinate systems

**Fig. 6:**
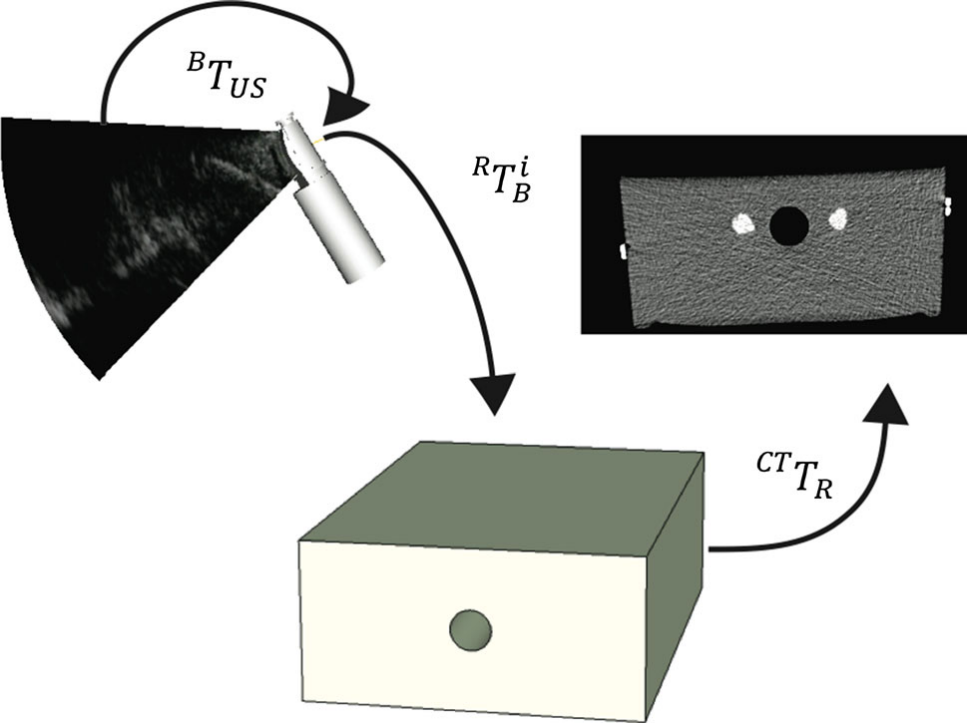
Transformation between the coordinate systems of the ultrasound image (US), the positions sensor on the bronchoscope (B), the reference sensor (R) on the phantom, and the computer tomography image of the phantom (CT)

The transformations between the coordinate systems described in this paper are illustrated in Fig. 6. A reference position sensor *R* was attached to the phantom, and another position sensor *B* was mounted close to the tip of the EBUS bronchoscope. The tracking system reported the position of sensor *B* relative to the reference sensor *R*, i.e., the transformation ${}^{R} T_{B}^{i}$ from *R* to *B* at time *i*. The probe calibration, i.e., the transformation from the US image coordinate system to the EBUS sensor coordinate system, is denoted as ${}^{B} T_\mathrm{US}$. The image registration is the relation between the coordinate systems of the sensor on the phantom and the CT images, ${}^\mathrm{CT} T_R$. A position in the ultrasound image in the CT coordinate system is found by:

$$\begin{aligned} p_\mathrm{CT}^i = { }^\mathrm{CT} T_R \times { }^R T_B^i \times { }^B T_\mathrm{US} \times p_\mathrm{US}^i \end{aligned}$$

where $p_\mathrm{US}^i$ is the position in the ultrasound image coordinate system, i.e., the origin [0 0 0 1].

### Registration

An image registration matches the CT image data to the patient at the start of the bronchoscopy (Figs. 2, 6). Both the 3D CT image volume and EM navigation field have excellent spatial accuracy, but need the alignment process to find the rotation and translation between the two physical spaces. The resulting registration matrix, ${ }^\mathrm{CT} T_R$ in Fig. 6, enables the navigation system to convert the tracked instrument positions in the navigation field to corresponding positions in the CT image volume. The positions of the fiducials in the coordinate system of the phantom reference sensor (P) were found by using an electromagnetic tracking (EMT) pointer. The fiducials were also pinpointed in the CT image volume. The registration matrix was obtained by using a closed-form method that minimizes the total distance between the corresponding points in the CT image and the coordinate system of the phantom reference.

CustusX also has a built-in automatic image-to-patient centerline-based registration method [38], but this would not provide adequate registration accuracy due to the very simplified phantom airway model in this study [38].

### EBUS probe calibration

**Fig. 7:**
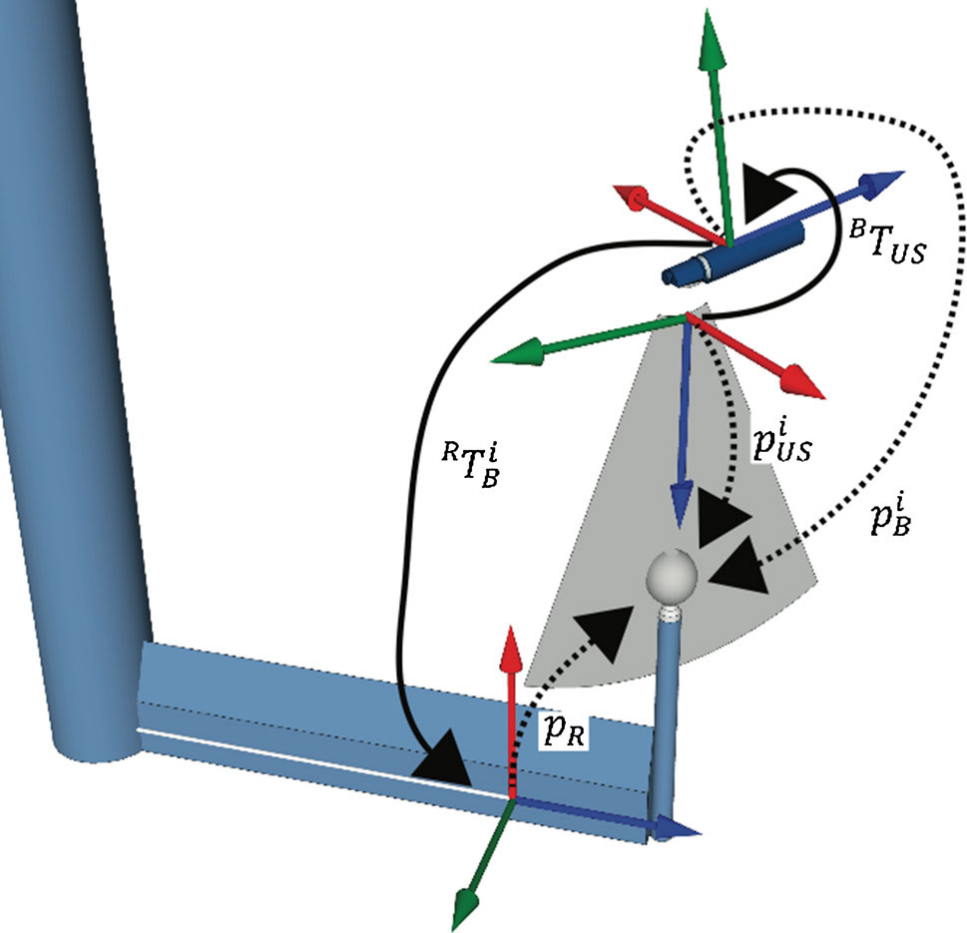
Imaging target is moved through the ultrasound (US) image plane. $p_{R} , p_{B}^i\hbox { and }p_\mathrm{US}^i$ describes the coordinate vectors of the image target relative to the sensor on the calibration arm, the sensor on the convex probe endobronchial ultrasound (CP-EBUS) bronchoscope and the US image plane. ${}^{B} T_\mathrm{US}$ and ${}^{R} T_{B}^i$ are matrices describing the transformation from the US image plane to the sensor on the EBUS probe and from the sensor on the CP-EBUS probe to the sensor on the calibration arm

In order to find the spatial relation between the real-time US image and the EMT sensor on the EBUS probe, a probe calibration [40] had to be performed. The basic principle of most probe calibration methods is to image an object whose position is known in the tracking coordinate system. We imaged a small plastic sphere (diameter 11.5 mm) mounted on a calibration arm, as illustrated in Fig. 7. A reference EMT sensor *R* was attached to the calibration arm, and the position of the center of the sphere $$p_R $$ relative to the reference sensor was measured prior to the calibration procedure using the tracking system.

The calibration arm was then attached to a six-axis robot arm (UR5, Universal Robots, Odense, Denmark) and submerged in water. The EBUS probe was located in a fixed position directly above the imaging target. The robot arm then moved the plastic sphere slowly (1 mm/s), first through the ultrasound image plane and then in the opposite direction along the same path, resulting in two sequences of recorded US images of the sphere passing through the US plane. This was repeated for nine positions evenly distributed throughout the image plane. For each US image, the transformation ${ }^R T_B^i$ representing the position and orientation of the US probe relative to the reference sensor on the calibration arm was acquired.

**Fig. 8:**
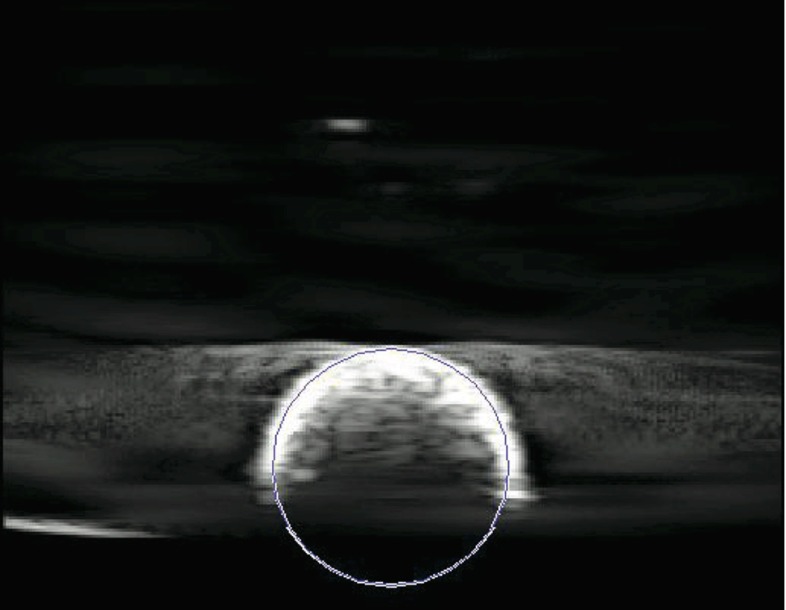
An ultrasound image cutting through the imaging target (plastic sphere) during probe calibration. The location of the sphere center within that image was found by adjusting the position of a virtual circle of the same diameter as the sphere, until its circumference corresponded with the surface of the imaged sphere

Next, the position of the plastic sphere within each of the totally 18 US recordings was manually located. Each recording was read into the software MATLAB (MathWorks, Natick, MA, USA) and displayed. The image within the recording where the center of the sphere passed through the US image was found. Furthermore, the location of the sphere center within that image was found by adjusting the position of a virtual circle of the same diameter as the sphere, until its circumference corresponded with the surface of the imaged sphere (Fig. 8). Thus, the sphere center position in each of the 18 US recordings was found to be $p_\mathrm{US}^i$, for $i = 1, \ldots , 18$.

The corresponding transformation $${ }^R T_B^i $$ was then extracted from the tracking data. Combining this information with the previously measured position of the image target $$p_R $$ relative to the reference on the calibration arm, the imaging target’s position relative to the position sensor on the EBUS probe was found as:

$$\begin{aligned} p_B^i ={ }^B T_R^i \times p_R =\left( {{ }^R T_B^i } \right) ^{-1}\times p_R \end{aligned}$$

The calibration matrix $${ }^B T_\mathrm{US}$$ was obtained by using a closed-form method that minimizes the total distance between the two corresponding sets describing the 18 positions of the image target: in the US image coordinate system $$p_\mathrm{US}^i $$ and in the EBUS probe’s coordinate system $$p_B^i $$. A more thorough description of the probe calibration procedure can be found in Bø et al. [41].

### Experiment

The experiment was performed in the bronchoscopy suite at the Dep. of Thoracic medicine, using the in-house prototype EBUS bronchoscope with an external EMT sensor. The experimental setup is illustrated in more detail in Fig. 4.

#### Navigated EBUS functionality

**Fig. 9:**
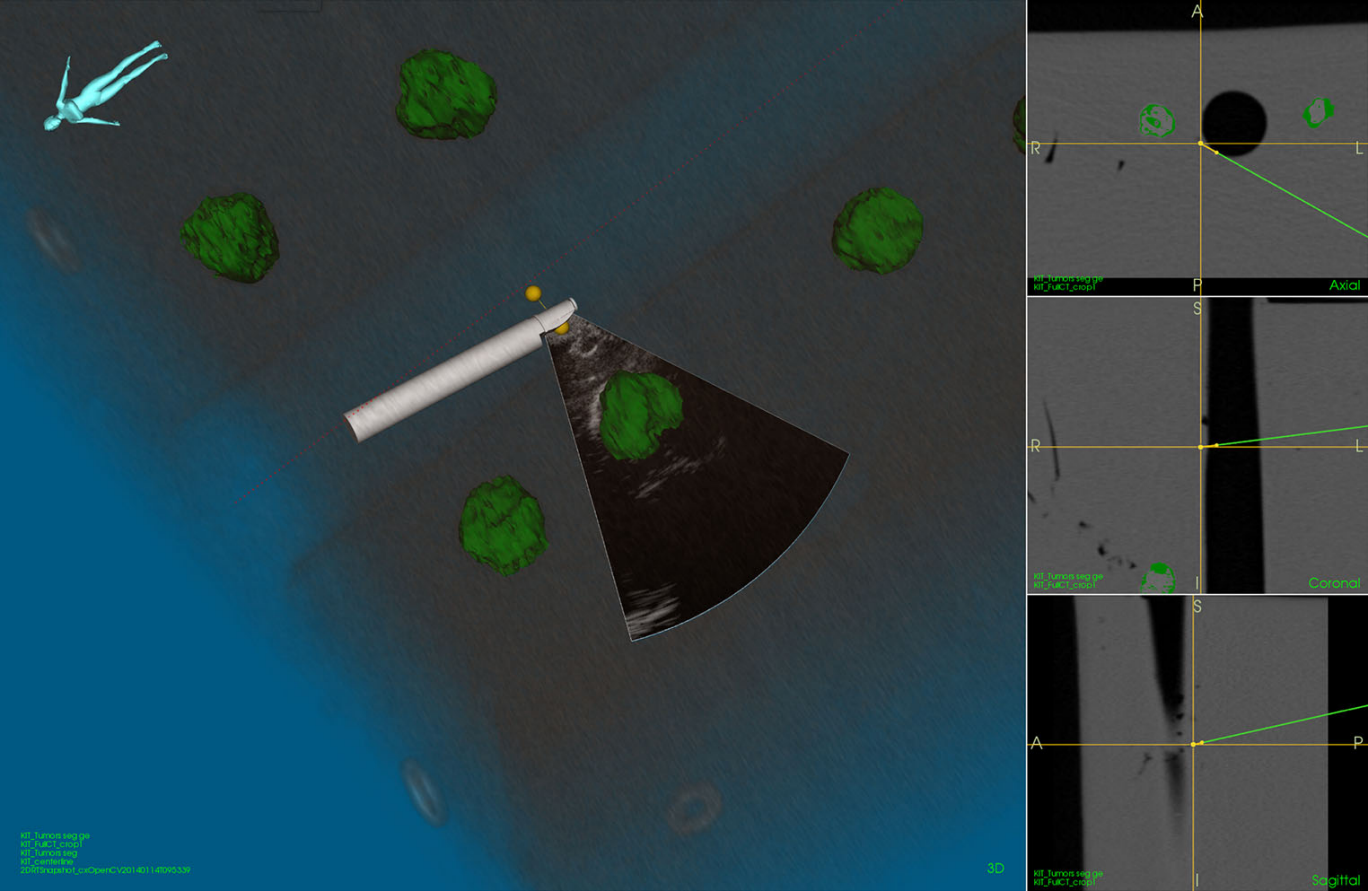
Graphical user interface (GUI) of navigation system during tracked convex probe endobronchial ultrasound (CP-EBUS) in a phantom. A model of the tip of the EBUS bronchoscope and the real-time ultrasound image are displayed. Tumor models are segmented from computed tomography (CT) (*green*). Axial, coronal, and sagittal views (*right side*). *Yellow crosshairs* top center position of ultrasound image. The 3D scene view direction of the patient/phantom is displayed in the *top left corner*

**Fig. 10:**
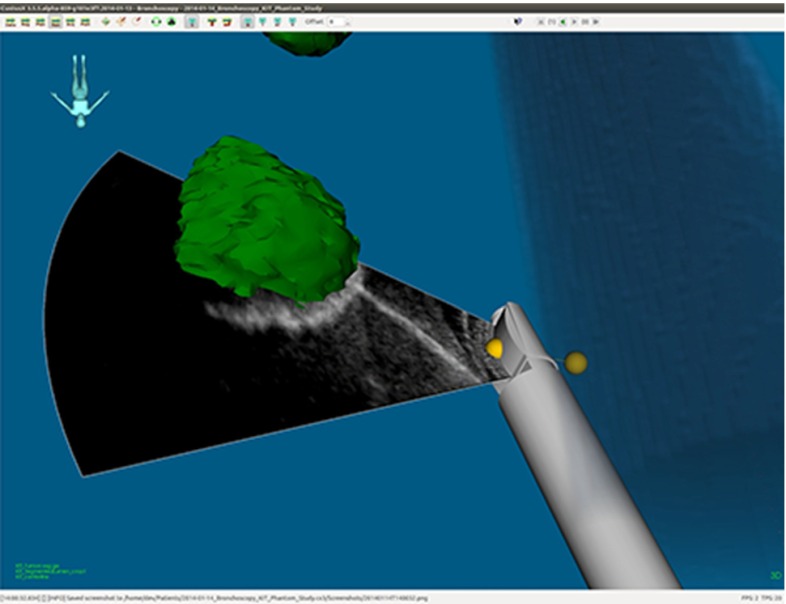
Navigated convex probe endobronchial ultrasound (CP-EBUS) graphical user interface (GUI) example, with ongoing fine-needle puncture of a target lesion. The fine needle is not tracked, but can easily be visualized sonographically

The EBUS bronchoscope was introduced into the phantom airways (Fig. 4). Conventional video bronchoscopy was used for orienting the tip of the EBUS bronchoscope in anatomical relation to the targets. The position of all 11 extraluminal targets was localized guided by EM navigation and confirmed by EBUS. The intraoperative position of the EBUS bronchoscope, corresponding preoperative CT images, real-time 2D US images, and segmented targets was displayed simultaneously in the navigation scene (Figs. 4, 9, 10).

#### Tracking accuracy

The EBUS bronchoscope was advanced through the phantom airway from the proximal to the distal end. EM bronchoscopy guided the localization and subsequent 2D US visualization of all 11 targets in the following order: 2R, 2L, 4R, 4L, 7, 10L, 11R. The experiment was repeated three times in the same sequence.

**Fig. 11:**
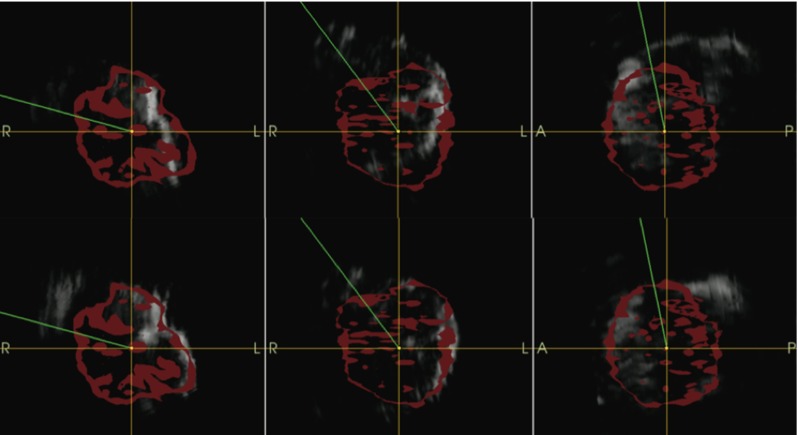
Manual shift correction to determine the position deviation between computed tomography (CT) and ultrasound (US) volumes. Reconstructed US volumes (*gray*) were moved manually to the corresponding surface model segmented from CT (*red*). The optimal alignment was then determined in 2D axial, coronal, and sagittal (ACS) planes. *Top row* ACS planes before manual correction. *Bottom row* ACS planes after manual correction

After each US acquisition, the inaccuracy of the system was visible in the navigation scene by the mismatch between the surface of the tumor model in the reconstructed US volume and the segmentation of the same model from the CT image. To quantify the system accuracy, we performed a manual shift correction of US volumes to overlay the segmentation of the model from CT. The alignment was found by using 2D ACS projections of the US and CT volume, and finding the optimal match of the target surface in all these planes (Fig. 11). The manual shift determined the position deviation between US and CT volumes, thus the system accuracy. One separate shift correction was found for each acquired US volume. The center position coordinates of all targets in the CT volume were pinpointed in ACS planes by an engineer experienced in image processing. The target center position in US was then calculated by adding the deviation found by the manual alignment.

## Results

### Navigated EBUS functionality

**Fig. 12:**
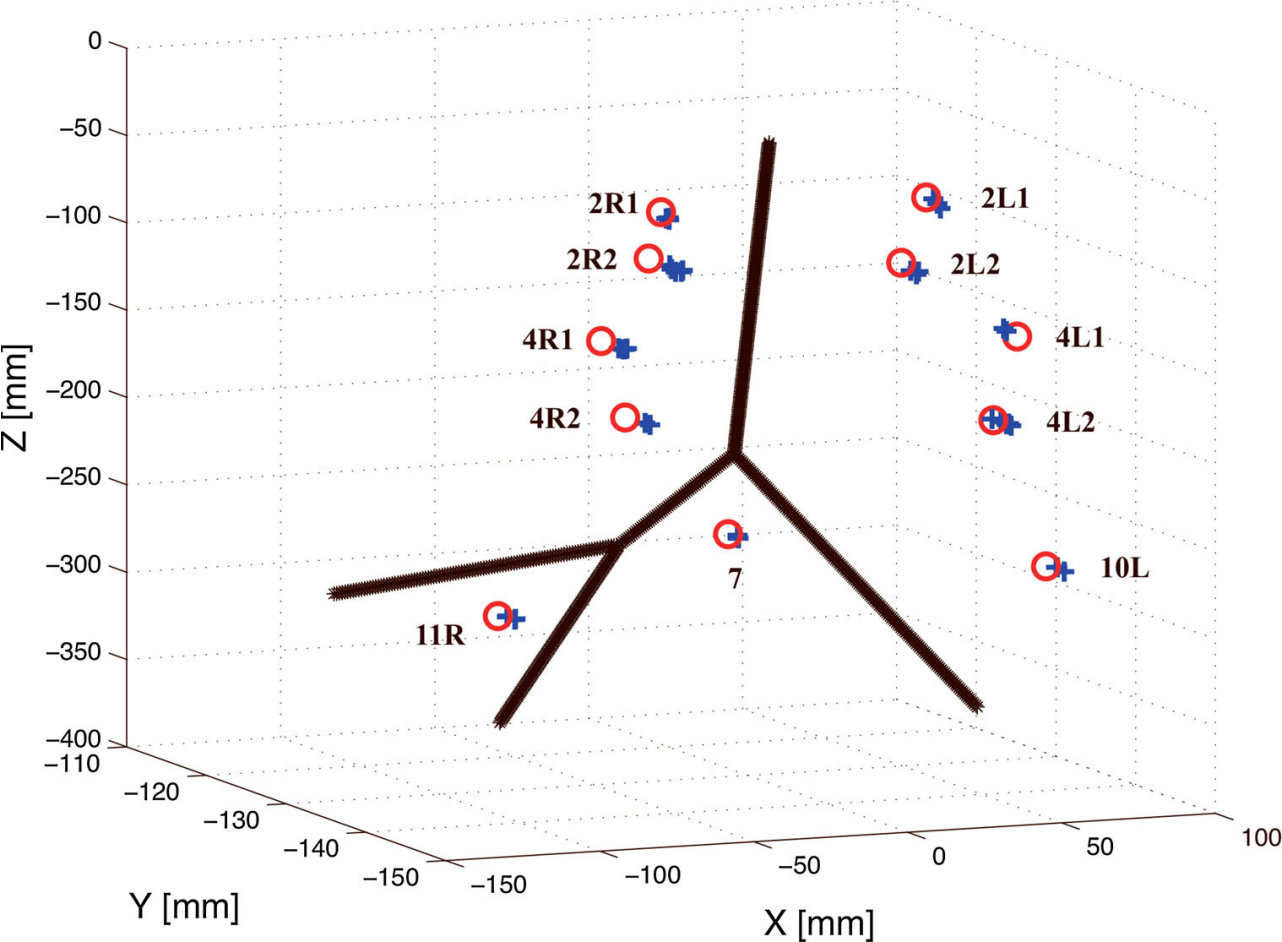
Position of 3D data acquired during navigated convex probe endobronchial ultrasound (CP-EBUS) in a phantom model. *Black line* centerline of airways extracted from computed tomography (CT). *Red circles* center position of tumor model in CT. *Blue crosses* center position of tumor model in ultrasound (calculated as center position in CT + deviation found by manual alignment)

Maneuvering the bronchoscope by combined US–CT–EM guidance was easy, fast, and corresponded well with the operator’s sense of correct bronchoscope localization. The traceable US bronchoscope had no limitations regarding maneuverability compared to the conventional EBUS bronchoscope, and the phantom model worked well for the feasibility experiment. Electromagnetic tracking of the EBUS bronchoscope and all registration algorithms worked smoothly and as intended. All targets were visualized, and their positions acquired and stored in the navigation system (Figs. 9, 10). The navigated EBUS system guided the operator directly and precisely to target, without adding time or complexity to the procedure. The intraoperative position coordinates of the EBUS bronchoscope and US images were stored in CustusX and could be retrieved after the procedure for documentation (Figs. 9, 10, 11, 12).

### Tracking accuracy

In total, 37 position measurements from 11 target lesions were taken (Table 1). Four targets had data acquisitions from more than three measurements, reflecting intraoperative doubt of whether the previous dataset was completely acquired for that target. Two targets had less than three measurements (Table 1), which was unintentional. Mean error observed in target lesion position between ultrasound and CT was 2.8 $\pm$ 1.0 mm, maximum error 5.9 mm. Maximum error was found in the 2R2 position, minimum error in position 7 (Fig. 12; Table 1). The position error for right-sided targets tended to be larger than the left-sided (Fig. 12; Table 1). Position data and error for all tumor models are presented in Table 1 and visualized in Fig. 12.

**Table 1:** Range of position coordinates for 11 target lesions in computed tomography volumes in the *x*-, *y*-, and *z*-planes

Tumor position	Number of measurements	Deviation in CT coordinates			Error
		*X*	*Y*	*Z*	
2R1	3	0.9 to 1.2	$-$0.7 to ($-$0.2)	$-$3.6 to ($-$2.6)	2.8 to 3.8
2R2	5	1.2 to 3.3	$-$3.4 to ($-$1.9)	$-$4.5 to ($-$1.0)	2.9 to 5.9
2L1	3	$-$0.3 to 0.5	$-$1.9 to ($-$0.5)	$-$3.3 to ($-$0.2)	1.2 to 4.2
2L2	3	$-$0.5 to 0.0	$-$2.1 to ($-$1.2)	$-$3.1 to ($-$1.6)	2.6 to 3.8
4R1	4	1.4 to 1.7	$-$2.6 to ($-$1.7)	$-$1.6 to ($-$0.3)	2.7 to 3.1
4R2	2	1.4 to 1.6	$-$2.6 to ($-$1.9)	$-$0.2 to ($-$0.2)	2.5 to 2.9
4L1	2	2.8 to 2.9	2.4 to 2.8	$-$1.2 to ($-$0.3)	3.9 to 4.0
4L2	5	1.9 to 2.1	$-$1.4-0.9	$-$1.0 to ($-$0.2)	2.0 to 2.7
7	3	$-$0.2 to 0.5	$-$1.4 to ($-$1.0)	$-$0.2 to 1.0	1.2 to 1.6
10L	3	1.0 to 1.4	$-$1.9 to ($-$0.9)	0.0 to 0.9	1.6 to 2.2
11R	4	1.9 to 2.6	$-$1.4 to ($-$0.2)	$-$0.2 to 0.0	2.3 to 2.9
*n*	37				
Mean		1.4	$-$1.2	$-$1.1	2.8
SD		0.9	1.3	1.3	1.0
Maximum					5.9

Deviation and error in millimeters

## Discussion

We have proved the feasibility of a novel EM navigated CP-EBUS system in a dedicated, in-house phantom model. In keeping with the intention of the experiment, we were able to track the endoluminal position of the CP-EBUS bronchoscope using EMT and fusion of real-time 2D US and preoperative CT volumes.

The study results provided a system accuracy estimate of 2.8 $\pm$ 1.0 mm. This includes potential errors from probe calibration, registration, the EM positioning system, and disturbances to the EM field. Since position data were measured repeatedly against several targets, we also got an impression of the robustness of the navigated CP-EBUS system, which seemed good (Table 1). The error component in the *z*-direction seemed smaller when imaging tumor models positioned in the peripheral parts of the phantom volume. This probably reflects an error component from the image registration, or external disturbances to the EM field.

The in-house airway phantom proved feasible for demonstration of the navigated CP-EBUS system functionality parameters: accuracy, robustness, and clinical usefulness. The traceable CP-EBUS bronchoscope was easy to operate and functioned to its purpose. The navigated CP-EBUS system was feasible for target localization and intraoperative position control of the bronchoscope. All phantom targets were successfully visualized in the 2D and 3D imaging scenes. The correspondence between US, visual control, and the navigation scene display was accurate, judged by the operator. All position coordinates and image documentation were stored in CustusX and available for retrieval. The store and retrieve ability of images and position data provided exact documentation of which targets have been visualized and/or sampled, and in what order.

The current study is a preclinical proof-of-principle experiment. To its advantage, the phantom model was simple, low cost, and stable enough for precision and robustness testing. Despite the lack of respiratory movement, we find our experimental model fairly realistic of a real-life setting. Even with modification, the traceable CP-EBUS bronchoscope was similar in size and functionality to the conventional CP-EBUS bronchoscope, and the positioning sensor is not expected to interfere with the clinical workflow. The navigation system utilizes regular, low-cost disposable TBNA needles from a commercial vendor. In addition to the EM navigation features, the GUI of our system displayed image views (CT, 2D US) already familiar to the operator during CP-EBUS-TBNA, increasing the user-friendliness of the multimodal image guiding.

The results presented in this paper have to be carefully interpreted, as they originate from one phantom experiment only. Real-life respiratory movement, heart beats, and disturbances in the EM field from other medical technical equipment could influence the navigated CP-EBUS system accuracy. In clinical cases, time constraints and unforeseen patient-related events can also challenge the workflow of any image-guiding system. Further validation studies are therefore needed before routine clinical use.

The manual shift correction from US to CT volumes for system accuracy calculations may have introduced error to the experiment, potentially avoided or minimized by letting several persons perform the shift correction. Another limitation to the experimental platform is that the TBNA needle is not traceable in the navigation system. As for now, the operator have to rely on US guidance during the target sampling situation, like in conventional CP-EBUS. By implementing tracked sampling tools, we could get a better impression of the usefulness of fused EM navigation and US during the sampling process, and hopefully increase TBNA precision. Traceable TBNA needles, which are disposable, would however be more costly than using only a permanently mounted position sensor on the bronchoscope.

As far as the research and development platform for navigated CP-EBUS is concerned, both software and hardware are in-house solutions. However, since the software recently has been made available through open source, reproducing the study results will be easier.

Some commercial systems involve a position tracking sensor within the working channel of the bronchoscope [24]. In our system, the EM sensor is mounted and sealed on the tip of the US bronchoscope, closely positioned to the US probe but not interfering with the US scan sector (Fig. 3). Leaving the working channel free keeps it open for other purposes (suction, tissue sampling, saline or anesthetic agent installation), and assessing navigation throughout the procedure is possible. The preferable solution would probably be an EM sensor embedded inside the bronchoscope, but outside the working channel. This solution has formerly been attempted in our navigation system, but proved technically difficult and time-consuming. Knowledge of the exact position and orientation of the EM sensor is required to perform a valid probe calibration. In navigated EBUS, the probe must therefore be permanently attached, either inside or on the outer surface of the EBUS bronchoscope. A loose EM probe in the working channel will not be adequate for this purpose. In this navigated CP-EBUS experiment, we opted for a simplified, external EM sensor, aiming for human testing where stability is crucial.

Although multimodal image guiding in bronchoscopy is increasingly called for by endoscopists, similar studies implying navigated EBUS (RP-EBUS or CP-EBUS) are scarce. The commercial systems previously combining EBUS and EM navigation in clinical studies applied sequential procedures with no real-time tracking of the EBUS images [24, 25]. These studies have utilized RP-EBUS miniprobes, and the clinical field of use was guiding TBNA of peripheral lung lesions. RP-EBUS should not be confused with the CP-EBUS probe used in our system, as RP-EBUS rarely is used to target mediastinal lymph nodes in lung cancer staging. The results from studies using commercial navigation equipment in the lung periphery can therefore for several reasons not be applied to our R&D platform.

The image-guiding system of Zang et al is, like our navigated CP-EBUS system, addressing the problem of 2D EBUS not being directly linked to preoperative CT images. Zang’s system does however require an extensive amount of planning and manual image preprocessing, probably making it more suitable for postoperative analysis and learning purposes. Luo and Mori’s method for navigated EBUS distinguishes from ours in several aspects. They use an RP-EBUS probe, with a different clinical field of use than the CP-EBUS bronchoscope. Tracking of the radial probe is possible due to an external sensor that could be vulnerable to mechanical strain intraoperatively. The attachment site of the sensor could possibly interfere with the US image, which is an important part of the diagnostic tool for the operator. Neither Luo’s system nor ours have traceable sampling tools.

The main improvement over existing solutions is that our system allows actual guiding of a convex probe EBUS bronchoscope designed for transbronchial lymph node sampling in the mediastinum. Even when bronchoscope vision is reduced due to blood or mucus within the airways, the combined CP-EBUS and preoperative CT in the same image provides concurrent, real-time image-guided position control and needle puncture. Our navigated CP-EBUS system also makes recording of US imaging combined with the equivalent CT slice possible for documentation purposes. This is not available in current EBUS-TBNA systems. The endoscopist can thereby replay the entire navigated procedure, ensuring that the correct lymph nodes are sampled and in the correct sequence. This is important in lung cancer staging, where mediastinal lymph node sampling should be carefully planned and evaluated to avoid errors depriving patients of life-saving surgery.

All our technological solutions are focused on user-friendliness and availability through open-source access, and imminent implementation in the clinic is a paramount aim. Basically, anyone can perform EM navigated EBUS with an available EBUS probe and an Aurora or similar EM sensor. The commercial providers currently do not sell their systems with EM-based tracking for any type of EBUS.

CP-EBUS-TBNA is now the method of choice for mediastinal lung cancer staging in all patients with potential to cure [7]. Intraoperatively, CP-EBUS can visualize small lymph nodes not detected in preoperative CT or PET–CT. These may also contain malignant tissue and should consequently be sampled to avoid futile thoracic surgery [7]. Precise, accurate and minimally invasive lung cancer staging procedures will be highly demanded, and introducing a navigation system for CP-EBUS-TBNA is therefore provident research preparing for future clinical requirements.

Improving a successful technique such as CP-EBUS is indeed a challenge. Like many other developments in diagnostic or surgical procedures, the navigated CP-EBUS system presented might not be able to improve success rates. Hopefully, the new approach can increase diagnostic confidence in closely located lymph node stations, where puncturing the wrong lymph node will have important clinical implications. Locating smaller lymph nodes in technically challenging positions could be easier with combined CP-EBUS/navigation technology. Fast and easy target localization, reproducible sampling, and improved quality control could also minimize the inter-operator performance variability.

The clinical use of CP-EBUS-TBNA will become more widespread and challenging in the future lung cancer work-up due to international agreement on routine systematic mapping [7]. Strategies to reduce procedure time, improve procedure safety and (3D) documentation, and develop learning tools for endoscopists will therefore be equally important. Our navigated CP-EBUS system may contribute to all of these aspects.

Navigated CP-EBUS seems promising for increased efficiency throughout the lung cancer diagnostic patient course, potentially saving time and health resources in line with governmental areas of priority. Traceable TBNA needles may increase the diagnostic reach in the mediastinum, and together with 3D US provide more detailed overview and needle position control than by 2D US guidance alone. The image acquisition store and replay functionality is interesting for medical record procedure documentation, interventional training, multidisciplinary meetings, and therapeutic decision making. Future challenges in refining the navigated CP-EBUS system are improved integration of sensors into the instruments and sampling tools, correction of errors from respiratory movement, and refining visualization techniques and software to better suit the procedure workflow.

## Conclusion

A CP-EBUS navigation system is needed to meet future requirements of lung cancer staging in the mediastinum. Our novel platform for EM navigated CP-EBUS performed well in a lung phantom concerning basic functionality, image fusion, target localization, robustness, accuracy, and user-friendliness. Even though some technological solutions must be refined, the EM navigated CP-EBUS system is ready to be applied in the clinic. Human studies are ongoing.
